# Understanding Host–Pathogen Interactions in Congenital Chagas Disease Through Transcriptomic Approaches

**DOI:** 10.3390/pathogens14020106

**Published:** 2025-01-22

**Authors:** Tatiana M. Cáceres, Luz Helena Patiño, Juan David Ramírez

**Affiliations:** 1 Centro de Investigaciones en Microbiología y Biotecnología-UR (CIMBIUR), Facultad de Ciencias Naturales, Universidad del Rosario, Bogotá 111221, Colombia; tatiana.caceres@urosario.edu.co (T.M.C.); luzh.patino@urosario.edu.co (L.H.P.); 2 Molecular Microbiology Laboratory, Department of Pathology, Molecular and Cell-Based Medicine, Icahn School of Medicine at Mount Sinai, New York, NY 10029, USA

**Keywords:** congenital Chagas disease, transcriptomics, host–pathogen, *Trypanosoma cruzi*

## Abstract

Chagas disease, caused by *Trypanosoma cruzi*, is a parasitic zoonosis with significant health impacts, particularly in Latin America. While traditionally associated with vector-borne transmission, increased migration has expanded its reach into urban and non-endemic regions. Congenital transmission has become a critical route of infection, involving intricate maternal–fetal immune interactions that challenge diagnosis and treatment. This review synthesizes findings from three RNA-seq studies that explore the molecular underpinnings of congenital Chagas disease, emphasizing differentially expressed genes (DEGs) implicated in host–pathogen interactions. The DAVID tool analysis highlighted the overexpression of genes associated with the innate immune response, including pro-inflammatory cytokines that drive chemotaxis and neutrophil activation. Additionally, calcium-dependent pathways critical for parasite invasion were modulated. *T. cruzi* exploits the maternal–fetal immune axis to establish a tolerogenic environment conducive to congenital transmission. Alterations in placental angiogenesis, cellular regeneration, and metabolic processes further demonstrate the parasite’s ability to manipulate host responses for its survival and persistence. These findings underscore the complex interplay between the host and pathogen that facilitates disease progression. Future research integrating transcriptomic, proteomic, and metabolomic approaches is essential to unravel the molecular mechanisms underlying congenital Chagas disease, with a particular focus on the contributions of genetic diversity and non-coding RNAs in immune evasion and disease pathogenesis.

## 1. Introduction

Chagas disease (CD), caused by the hemoflagellate protozoan *Trypanosoma cruzi* (*T. cruzi*), is a parasitic zoonosis with significant implications for global public health. The World Health Organization (WHO) identifies it as one of the 20 neglected tropical diseases, with a prevalence estimated at 6 to 7 million cases across 21 of the 33 countries in Latin America [1]. Historically, the geographical distribution of CD was predominantly rural, where vector-borne transmission via hematophagous triatomine insects was the principal route of infection. However, anthropogenic factors such as climate change, ecosystem fragmentation, and urbanization have facilitated its spread into urban centers and non-endemic regions, altering its epidemiological pattern.

In addition to vectorial transmission, *T. cruzi* exhibits a capacity for dissemination through non-vectorial mechanisms, including blood transfusions, organ transplantation, ingestion of contaminated food, and accidental exposure in laboratory settings. Among these routes, congenital transmission—from infected mothers to their offspring—has garnered increased attention as a critical pathway contributing to the disease burden [2,3,4]. This mechanism, exacerbated by the migration of infected populations to non-endemic regions, poses significant diagnostic and therapeutic challenges in countries with limited resources for prenatal and neonatal screening [5,6].

To reduce transmission, vector control programs and blood and organ screening have been implemented in endemic areas, significantly decreasing vector-borne and transfusional transmission. However, congenital transmission remains a major source of new cases. This mechanism presents considerable challenges, mainly due to the difficulty in accurately estimating its global burden, compounded by the lack of representative epidemiological data in many regions and the absence of systematic record-keeping. Current estimates suggest that morbidity is 28.3%, while mortality among affected neonates is 2.2%. These figures likely underestimate the true scope of the problem [7].

Congenital transmission of *T. cruzi* is a complex process involving significant maternal and fetal immune system modulation. Several factors influence the risk of congenital transmission, including maternal factors such as immune status and obstetric history, as well as parasite-related factors such as strain and parasitic load. Some mothers may transmit the parasite in one or more pregnancies, while others do not, and the reasons for this variability are not yet fully understood [8]. During pregnancy, the physiological immunosuppression that prevents fetal rejection may promote the reactivation of parasitemia, as indicated by elevated levels of *T. cruzi*-specific IgM [9]. Mothers who do not transmit the parasite typically exhibit a more pronounced immune response, characterized by elevated levels of proinflammatory cytokines such as TNF-α, IL-1β, and IL-6, which are also detected in their uninfected neonates. In contrast, mothers of neonates with congenital infection demonstrate reduced levels of TNF and IFN-γ, along with diminished expression of key antigen-presenting molecules such as HLA-DR and CD54 [8,10]. These findings underscore the critical role of maternal immune regulation in vertical transmission, highlighting the need for further research to better understand and address this complex process. The parasite employs multiple strategies to invade host cells and evade immune responses, complicating accurate and timely diagnosis and treatment. Furthermore, the use of teratogenic drugs like benznidazole and nifurtimox during pregnancy is contraindicated, allowing neonatal infection to progress to chronic stages of the disease. Consequently, these chronic forms can lead to severe complications, including cardiopathies and gastrointestinal disorders in adulthood [11].

Traditional approaches to studying congenital Chagas disease (CD) are constrained in their ability to elucidate the complex mechanisms underpinning vertical transmission. In contrast, transcriptomics has emerged as a robust and high-resolution tool for analyzing the genetic and molecular dynamics of host–pathogen interactions. This approach allows for the identification of biomarkers and pathways crucial to understanding disease mechanisms and improving diagnostic and therapeutic strategies [12,13]. The utility of transcriptomics is well demonstrated in infectious diseases like tuberculosis, where it has been instrumental in identifying diagnostic markers and differentiating infection states. For instance, the METTL7B gene distinguishes active tuberculosis from latent infection, while the HBA1/2 and HBD genes differentiate *Mycobacterium tuberculosis* from *M. avium* [14]. In respiratory conditions such as pneumonia and chronic obstructive pulmonary disease (COPD) exacerbations, transcriptomic studies have identified over 1600 differentially expressed genes related to inflammation and immune response, advancing diagnostic accuracy and personalized treatment [15]. In the context of congenital CD, transcriptomic approaches offer a framework for investigating critical factors influencing transmission, such as maternal parasitemia, *T. cruzi* genetic diversity, placental molecular interactions, and immune system modulation. By integrating transcriptomic data into multidimensional analyses, researchers can achieve a more comprehensive understanding of the host–pathogen interface [16]. This integration holds promise for refining early diagnostic protocols and developing targeted interventions to mitigate the burden of congenital transmission.

Recent advances in transcriptomic studies have profoundly enriched our understanding of the biological mechanisms driving congenital transmission of *Trypanosoma cruzi*. However, significant knowledge gaps persist regarding the primary mechanisms and immunoregulatory factors that influence fetal infection. Investigations into congenital Chagas disease have employed diverse models, focusing on key pathways such as NF-κB and Toll-like receptor (TLR) signaling, alongside evaluations of placental barrier integrity and its role in parasite invasion [17,18]. Despite these efforts, faithfully replicating the intricate architecture and functional dynamics of the human placenta in experimental systems continues to be a formidable challenge, limiting our ability to fully elucidate the molecular and cellular processes involved. This review provides a structured and comprehensive overview of congenital Chagas disease, beginning with the life cycle and genetic diversity of *Trypanosoma cruzi* and their implications for transmission and pathogenesis. It also examines the mechanisms of interaction between *T. cruzi* and the placenta, setting the stage for a deeper analysis of trophoblast infections. The primary objective is to leverage transcriptomic analyses to identify and characterize the critical biological processes, molecular pathways, and cellular mechanisms affected during infection, with particular emphasis on elucidating the host–pathogen interactions that facilitate congenital transmission.

## 2. Life Cycle and Genetic Diversity of *T. cruzi*: Implications for Transmission and Pathogenesis

**Figure 1:**
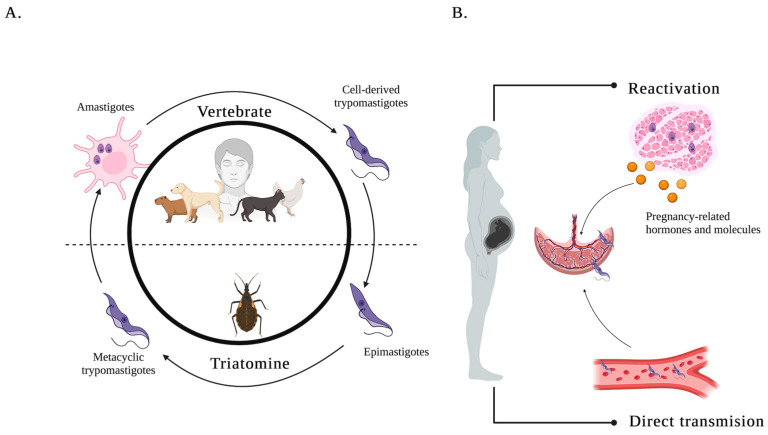
Life cycle. (**A**) The life cycle of *T. cruzi* alternates between vertebrate hosts and the triatomine insect vector. In vertebrates, metacyclic trypomastigotes invade host cells, where they differentiate into intracellular amastigotes, the replicative stage. Amastigotes multiply and transform into cell-derived trypomastigotes, which are released into the bloodstream to infect new cells or be ingested by a triatomine during a blood meal. In the triatomine, trypomastigotes differentiate into epimastigotes in the midgut, where they replicate and eventually transform into infective metacyclic trypomastigotes in the rectal ampoule, completing the cycle. (**B**) Mechanisms of congenital transmission of *T. cruzi.* During pregnancy, *T. cruzi* can be transmitted to the fetus through two mechanisms: (1) reactivation of infection: pregnancy-related hormones stimulate amastigotes in maternal tissues to transform into cell-derived trypomastigotes (CDTs), which are released into the maternal bloodstream. These CDTs can cross the placenta, enter fetal circulation, and spread the infection to fetal organs. (2) Direct transmission: CDTs circulating in the maternal blood invade trophoblastic cells in the placenta, crossing the placental barrier to enter the fetal bloodstream and infecting fetal tissues.

The life cycle of *T. cruzi* is intricate and involves two primary hosts: an insect vector and a mammalian host. It begins when an infected insect vector feeds on the blood of a mammalian host, simultaneously defecating and releasing metacyclic trypomastigotes (MTs), the infective form of the parasite, into its feces. The MTs enter the host through the wound caused by the insect bite, typically through mechanical transmission during scratching. Once inside the host, the MTs invade cells near the entry site and transform into amastigotes. Within these host cells, amastigotes actively proliferate, and after several rounds of replication, they differentiate into Cell-derived trypomastigotes (CDTs), which are then released into the bloodstream [19,20,21] (Figure 1).

This complex life cycle involves multiple stages, each of which plays a crucial role in the parasite’s ability to persist, replicate, and transmit between hosts. The genetic diversity of *T. cruzi* adds a layer of complexity, influencing the parasite’s ability to adapt to different environments and evade the immune response, and contributes to variations in disease outcomes across different regions and individuals. Understanding these processes is essential for unraveling the mechanisms underlying transmission and pathogenesis, particularly in congenital CD [22,23].

In an infected pregnant woman, there are two possible scenarios for the transmission of *T. cruzi* to the fetus: reactivation of the infection and direct transmission.

### 2.1. Reactivation of the Infection

Amastigotes present in maternal tissues can be stimulated by pregnancy-related hormones and molecules, leading to their transformation into cell-derived trypomastigotes (CDTs). This reactivation causes the release of CDTs into the maternal bloodstream. These CDTs can then reach the chorionic villi of the placenta, where they can enter the fetal circulation [24,25].

### 2.2. Direct Transmission

Circulating CDTs in the maternal blood may meet the chorionic villi, which are in the intervillous space of the placenta. Upon contact with these structures, the CDTs invade the trophoblastic cells that line the villi and cross the placental barrier to enter the fetal blood vessels. In fetal circulation, the CDTs infect nearby cells, where they transform back into amastigotes. During this replicative phase, the amastigotes multiply and are subsequently released as CDTs into the fetal bloodstream. From there, they migrate and spread the infection to various fetal organs and tissues [26,27].

This dual transmission mechanism underscores the complexity of *T. cruzi* interaction with the maternal–fetal immune environment, with significant implications for the pathogenesis of congenital CD.

### 2.3. Genetic Diversity

A prominent feature of *T. cruzi* is its remarkable plasticity, reflected in its extensive genetic variability and intense intraspecific phenotypic diversity. While it was long assumed that this diversity was solely the result of clonal reproduction, recent research has uncovered the occurrence of genetic recombination events *in vitro* and the presence of hybridization in natural populations [28]. These findings have fundamentally challenged the longstanding hypothesis of strict clonality, revealing a greater degree of genetic complexity in *T. cruzi*. This paradigm shift has necessitated a revision of the parasite’s nomenclature, leading to the adoption of a classification system that more accurately represents the phenotypic and genotypic diversity observed within its populations [29,30].

This framework introduced the concept of Discrete Typing Units (DTUs), defined as groups of populations that are more genetically like each other than any other group and are characterized by shared molecular markers. Six DTUs, labeled TcI through TcVI, have been identified and are widely distributed throughout the Americas [31,32]. These DTUs exhibit distinct variations in virulence factors, tissue tropism, distribution among vectors and reservoirs, host immune responses, and clinical manifestations. These differences highlight potential associations between specific traits and individual DTUs [33].

However, the correlation between the genetic diversity of *T. cruzi* and the clinical manifestation of the disease remains challenging to establish. While differential tissue tropism has been observed in some strains, other studies have failed to show a consistent relationship between the parasite’s genetics and the clinical forms of the disease [33,34]. This underscores the need to investigate the behavior of different isolates, particularly in co-infection contexts, as interactions between strains could influence the course of the infection [35]. Additionally, it is essential to develop tools that can assess genetic variability and gene expression within parasite populations to understand their role in pathogenesis better. Without a clear understanding of the mechanisms behind these differences, this area of research remains a critical focus in eco-epidemiology and clinical pathophysiology. The complexity of these interactions complicates the ability to generalize host interactions with different DTUs and establish a unified invasion model [36,37].

Genotyping studies on congenital *T. cruzi* transmission have identified several DTUs, including TcI, TcII, TcIII, TcV, and TcVI. Most of these studies have been conducted in endemic regions of South America, such as Argentina, Bolivia, and Chile, where the TcII, TcV, and TcVI genotypes are most common in both pregnant women and adults with chronic CD. Among these, TcV is particularly notable for its high prevalence, being found in 80–100% of congenital transmission cases in countries like Argentina, Bolivia, southern Brazil, Chile, and Paraguay. In contrast, TcI and TcII are less frequently observed in this context [36,38,39,40,41,42]. Notably, congenital *T. cruzi* infections, including severe and sometimes fatal cases, have also been reported in non-endemic countries such as Spain, Italy, the USA, Japan, and Switzerland [43,44,45,46,47,48,49,50,51]. These cases, however, likely represent only a fraction of the true burden, as the lack of systematic programs for detecting and treating congenital Chagas disease in non-endemic regions limits the recognition and management of these infections.

In a study by Antinori, congenital transmission was reported within a family cluster in Italy, originating from a Bolivian immigrant family. Genotyping identified the presence of TcII and TcV, highlighting the relevance of these DTUs in non-endemic contexts, particularly among migrant populations [52]. The identification of potential links between genetic diversity, transmission risk, and the progression of congenital CD is an emerging area of research. Although the distribution of DTUs varies by country, these associations may significantly influence transmission rates and the severity of congenital CD [53,54]. Moreover, the increasing number of congenital cases reported outside endemic areas underscores the importance of understanding how genetic diversity affects transmission dynamics in diverse epidemiological settings.

Few studies have examined the behavior of DTUs in experimental congenital infections, typically focusing on just one or two *T. cruzi* genotypes. To understand how the parasite’s intrinsic characteristics affect congenital transmission, it is crucial to study a wider range of strains that represent all DTUs, considering their diverse biological properties. Some studies have revealed significant genetic variability within the same DTU, which is associated with congenital transmission. For example, Burgos and colleagues identified distinct minicircle signatures in *T. cruzi* strains from the same lineage in the Chaco province of Argentina [38]. Similarly, Herrera and colleagues identified mixed infections of TcI, TcII, and TcV in Argentina, Honduras, and Mexico. They also found a significant association between the parasite haplotypes and congenital transmission [55].

Furthermore, the interaction between *T. cruzi* subpopulations and the host significantly influences the course of congenital infection by modulating the immune response in tissues. Multiple studies have reported alterations in gene expression in the placenta during infection, underscoring the critical role of the local immune response in the placenta in determining the risk of congenital transmission [56,57].

It is essential to deepen our understanding of congenital *T. cruzi* transmission, a multifactorial process influenced by both parasite and host characteristics, which can lead to severe outcomes and high neonatal mortality rates. Strengthening global surveillance of this transmission route is a priority, as is expanding research to include a broader range of strains and geographical regions [35]. This will provide a more comprehensive understanding of the risk factors and help develop effective prevention and intervention strategies to reduce the burden of the disease.

## 3. Mechanisms of Interaction Between *T. cruzi* and the Placenta

The placenta is a temporary organ that connects the mother’s and fetus’s circulatory systems, exchanging nutrients and gases for fetal development. In addition to its physical connection role, the placenta produces hormones, peptides, and steroids that regulate various processes critical for the success of the pregnancy [18,58,59]. It also performs metabolic functions, modulates the maternal immune response to tolerate the fetus, and facilitates maternal–fetal communication. Importantly, the placenta serves as a protective barrier, preventing the transmission of pathogens and harmful molecules that could threaten fetal health [58,60]. This tissue consists of chorionic villi, which are in direct contact with maternal blood in the intervillous space. These villi are covered by two layers of trophoblastic cells: an outer layer of syncytiotrophoblasts (STs), a multinucleated, non-replicating layer, and an inner layer of cytotrophoblasts (CTs), a replicating cell layer [61]. Beneath the trophoblast lies the basal lamina and villous stroma (EV), which contains vascular endothelium, fibroblastic cells, and macrophages [62,63].

However, microorganisms such as *Toxoplasma gondii*, cytomegalovirus, rubella, herpesvirus, *Listeria monocytogenes*, *Treponema pallidum*, *T. cruzi*, and others have developed complex immune evasion mechanisms [64,65]. These pathogens possess a range of proteins and enzymes that allow them to bypass the placental barrier. They have evolved to disrupt placental functions, promoting their invasion and facilitating vertical transmission to the fetus. By modulating the immune response, destroying placental cells, and altering the physical and molecular barriers of the placenta, these pathogens can access the fetal environment, leading to severe complications, including malformations, developmental delays, or even fetal death [64,66]. The ability of these microorganisms to bypass placental protection emphasizes the complexity of congenital infections and highlights the need to understand the specific mechanisms of each pathogen. This knowledge is essential for developing more effective prevention and treatment strategies to reduce risks and improve outcomes for both mothers and affected fetuses.

The invasion of *T. cruzi* into host cells is a complex, multifaceted process involving dynamic interactions between the parasite and host cells. The parasite attaches to host cells through specific receptors on its surface. *T. cruzi* presents various molecules on its membrane that interact differently with host cell components and the extracellular matrix (ECM), aiding its invasion. During infection, the parasite is recognized by Toll-like receptors (TLRs) on host cells, which detect pathogen-associated molecular patterns (PAMPs). In the case of *T. cruzi*, several TLRs, including TLR-2, TLR-4, TLR-7, and TLR-9, are involved in this recognition. As these receptors are found in the chorionic villi of the placenta, they are believed to play a key role in the immune response to the infection, particularly in the congenital transmission of the parasite [67,68,69]. Upon pathogen recognition, trophoblastic cells trigger an innate immune response characterized by the release of cytokines, chemokines, reactive oxygen species (ROS), nitric oxide (NO), and antimicrobial peptides. In response to *T. cruzi* infection, a turnover of syncytiotrophoblasts is induced. This process involves the TLR-2 receptor, which subsequently activates caspase 8 and the MAPK ERK1/2 signaling pathway, leading to the elimination of the pathogen adhering to the surface cell layer [70].

Significant changes in the ECM have been observed in the placentas of women infected with *T. cruzi*. Histopathological findings include the destruction and detachment of syncytiotrophoblasts in chorionic villus explants, selective disorganization of the basal lamina, and alterations in type I collagen within the connective tissue [71,72]. Proteomic studies have shown that *T. cruzi* induces changes in the ECM by either increasing or decreasing the expression of ECM components through several mechanisms: (i) destruction of ECM components via protease activity, (ii) induction of matrix metalloproteinases (MMP-2 and MMP-9), (iii) modulation of signaling pathways, and (iv) reorganization of the cytoskeleton [63,73,74]. These events suggest that the reorganization of the ECM plays a crucial role in regulating inflammatory and immune responses in pregnant women. Together, these alterations contribute to the destabilization of the placental barrier, allowing the parasite to pass through to the fetus. Additionally, the presence of *T. cruzi* triggers an increase in human chorionic gonadotropin levels, which activates the MAPK ERK1/2 signaling pathway. This activation promotes cell proliferation and differentiation, potentially serving as a compensatory mechanism to help maintain the stability of the placental barrier [75].

**Figure 2:**
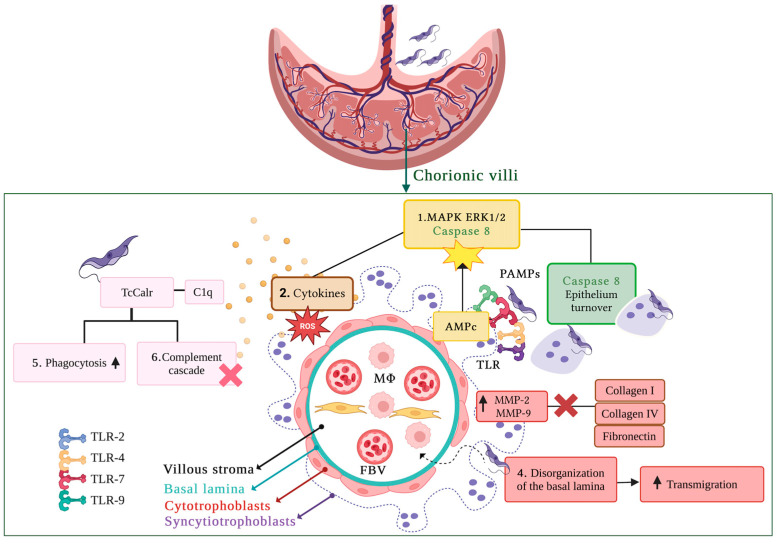
Host–pathogen interactions during congenital *T. cruzi* infection. This figure illustrates the interactions between the *T. cruzi* parasite and chorionic villi, emphasizing key molecular and cellular mechanisms: the interaction between surface molecules of *T. cruzi* and Toll-like receptors (TLRs) on trophoblastic cells initiates signaling cascades that increase cyclic AMP (cAMP) levels and activate the MAPK/ERK1/2 pathway (1). This pathway orchestrates multiple cellular responses, including the production of pro-inflammatory cytokines such as IL-6 and TNF-α, along with the generation of reactive oxygen species (ROS) (2). These inflammatory mediators exacerbate placental tissue damage and create a microenvironment conducive to parasite persistence. Additionally, *T. cruzi* infection triggers the activation of caspase-8, which facilitates the detachment of infected trophoblastic cells. This detachment contributes to cell turnover (3) and forms structural discontinuities in the placental barrier, allowing deeper parasite infiltration. The parasite also induces the overexpression of matrix metalloproteinases (MMP-2 and MMP-9), enzymes that degrade key components of the basal lamina, such as collagen types I and IV and fibronectin. This degradation disrupts the basal lamina’s structural integrity (4), enhancing parasite transmigration toward fetal tissues. Finally, *T. cruzi* calcireticulin (TcCRT) binds to C1q, a component of the complement system. This interaction promotes parasite opsonization, increasing its uptake by host cells (5). Concurrently, TcCRT disrupts the classical complement pathway, impairing the host’s immune response and facilitating parasite survival (6).

One of the key strategies used by *T. cruzi* to evade the host immune response is the expression of its pleiotropic protein, calreticulin (TcCalr), which is mainly localized in the flagellar region of the parasite. This protein recruits C1q, a component of the complement system that contains the dimeric serine proteases C1s and C1r. TcCalr’s primary function in this context is to prevent the activation of the complement cascade, thus aiding in immune evasion. Additionally, TcCalr acts as a molecular mimicry mechanism, as the TcCalr/C1q complex is recognized by host cells as a signal for phagocytosis, thereby increasing the parasite’s infectivity (Figure 2) [76]. This mechanism holds particular significance in the context of congenital transmission of CD, as elevated levels of calreticulin (Calr) have been observed during pregnancy. Furthermore, women who develop pre-eclampsia exhibit even higher levels of Calr compared to those without this complication. These findings underscore the critical role of calreticulin in the host–parasite interaction during congenital transmission of *T. cruzi* and suggest its potential as a biomarker for pre-eclampsia or as a therapeutic target [77,78].

The construction of specific transcriptional profiles is crucial for investigating the biological mechanisms underlying infectious disease development. This approach enables the large-scale analysis of dynamic changes in gene expression, allowing for the identification of thousands of genes while prioritizing those involved in critical processes such as cellular invasion, immune evasion, and disease progression [79]. The identification of differentially expressed genes (DEGs) is a critical first step in understanding how pathogens disrupt host systems. When combined with transcriptional profiling, pathway analysis offers a deeper understanding of the molecular and biological process alterations associated with disease. Tools such as Gene Set Enrichment Analysis (GSEA) and databases like KEGG and Reactome are essential for mapping these interactions. These integrated approaches not only pinpoint altered genes but also reveal the functional networks in which they are involved, providing insights into the mechanisms underlying inflammation, cellular stress responses, and tissue microenvironment remodeling [79,80].

These approaches have been essential for identifying biomarkers and proposing new therapeutic targets [81]. In the context of infectious diseases, genes involved in cytokine signaling, energy metabolism, and apoptotic pathways represent promising targets for developing personalized treatments. Integrating transcriptional profiles with pathway analyses not only enhances our understanding of the underlying pathological mechanisms but also supports the design of more targeted interventions. Additionally, combining transcriptomic data with other omics approaches, such as proteomics and metabolomics, provides a more comprehensive view of disease biology. This multidimensional approach opens new avenues in translational medicine, driving research toward innovative strategies for diagnosing and treating infectious diseases [82].

The construction of transcriptional profiles using advanced techniques such as microarrays and RNA-seq, followed by comprehensive bioinformatics analysis, enables the study of gene expression changes in the human placenta induced by interaction with the *T. cruzi* parasite. This approach not only facilitates the identification of molecular pathways involved in disease pathogenesis but also contributes to uncovering the specific mechanisms of congenital transmission and the host’s immune response to infection.

In this context, Castillo (2018) performed a transcriptional profiling analysis using microarrays on human placental explants infected ex vivo with *T. cruzi* (TcII). The analysis of differentially expressed genes (DEGs) revealed alterations in three main categories: (i) immune functions and inflammatory response, highlighting complement system components like CD46 and C1q, as well as the overexpression of Toll-like receptors (TLRs), including TLR-2, TLR-4, TLR-7, TLR-8, and TLR-9; (ii) extracellular matrix (ECM) remodeling, with up-regulation of genes such as ADAM12, ADAMTSL3, MMP10, MMP-2, and MMP-9; and (iii) placental development, nutrient transport, and hormonal function. The latter group represented the most overexpressed genes in infected tissues, including beta-1-glycoproteins, GH2, CSH1, and CSH2. These alterations may disrupt placental function, impairing the proper supply of nutrients and oxygen to the developing fetus, which could lead to complications such as growth restriction or pregnancy loss [56].

A subsequent study utilized RNA sequencing (RNA-seq) to analyze term placentas from both healthy women and women infected with *T. cruzi*, aiming to uncover the molecular mechanisms underlying placental dysfunction observed in pregnant women with chronic CD. Principal component analysis (PCA) revealed distinct clustering patterns based on serological infection status. Among the differentially expressed genes (DEGs), KIF12, a member of the kinesin superfamily involved in microtubule cytoskeleton dynamics, was the most prominently expressed transcript in the seropositive (SP) group. This was followed by HLA-G, which plays a key role in mediating immune tolerance during pregnancy, and PRG2, a cytotoxin involved in antiparasitic defense mechanisms and the inhibition of pregnancy-associated plasma protein A (PAPPA) [59]. Additionally, the seropositive group exhibited down-regulation of immune-modulatory elements, such as IL1F10 and kisspeptin (KISS1), both of which are involved in inhibiting chemotaxis and promoting the secretion of human chorionic gonadotropin (hCG). A negative regulation of hCG subunits, including CGB5, was also observed [83].

Several of the differentially expressed genes mentioned above have been previously linked to conditions such as pre-eclampsia, intrauterine growth restriction, spontaneous abortion, and recurrent pregnancy loss. These include the reduced expression of KISS1 and the subunits of human chorionic gonadotropin (CGB5), as well as the increased expression of neurokinin B (TAC3) and PAPPA [84,85]. Additionally, the dysregulation of glutamine and glutathione synthesis pathways observed in Juiz’s study aligns with the findings from the proteomic analysis of the human placenta conducted by Jin in 2017. This study highlights that not only the inflammatory response but also the dysfunction of glutathione metabolism contributes to the development of pre-eclampsia [83,86].

Considering the genetic diversity of *T. cruzi*, Juiz conducted a preliminary study to explore potential differences in host–pathogen interaction mechanisms in congenital CD across different parasite genotypes. The study aimed to examine how the TcI (K98, a myotropic clone) and TcVI (VD, a strain isolated from a congenital case) genotypes modulate the genetic response in the placental environment of mice during experimental chronic infection.

The results showed a higher number of differentially expressed genes (DEGs) in the TcVI genotype, with a greater tropism for placental tissue compared to the TcI genotype. However, functional analysis of the DEGs revealed no significant differences in the activation of innate immune pathways. On the other hand, cellular signaling analysis identified differential regulation in protein kinase and G protein-coupled receptor pathways, suggesting that the negative regulation of these pathways may be linked to the lower infection levels observed in the K98 group [57].

Faral and colleagues evaluated transcriptional and histological changes in placentas infected with congenital isolates (VT), medium-virulence strains (MV), and high-virulence strains (HV) of *T. cruzi*. The VT strains exhibited nearly undetectable parasitemia in BALB/cJ mice and 100% survival, indicating a silent phenotype and low virulence. In contrast, HV strains caused the highest parasitemia, reaching the endpoint of infection in all mice around day 19 post-infection. Despite differences in parasitemia and lethality, VT strains reached the same organs as HV strains and generated a similar immune response, primarily Th1, with the production of IFN-γ, IL-12, TNF-α, and IL-6.

Regarding congenital transmission, the MV strain failed to cross the placenta, while HV strains, although transmitted, did so at lower levels than VT strains. Additionally, HV strains altered the regulation of pregnancy-specific glycoproteins, reducing their levels in the placenta, a change not observed with VT strains. This finding was confirmed histologically, as placentas infected with HV strains showed greater tissue damage compared to those infected with VT strains. In terms of transcriptional profiles, there was a significant disparity in the number of differentially expressed genes (DEGs), with the HV strain showing greater regulation, with 2507 DEGs compared to 408 DEGs in the VT strains. Functionally, the HV strain exhibited overexpression of elements related to inflammation, cellular immune response, and ribosomal proteins, while the VT strains showed down-regulation of processes such as mitosis, the cell cycle, DNA replication, and the DNA damage response.

Based on these findings, the researchers propose two possible scenarios for congenital transmission: (i) exacerbated changes in placental gene expression related to inflammation, as seen in the HV strain, and (ii) induction of an immune response that does not harm the host or interfere with placental function, as seen in the VT strains. These scenarios reflect distinct invasion strategies, with congenital isolates being more efficient due to their low replication and high placental tropism. Although HV strains manage to cross the placental barrier, this process may be associated with tissue damage caused by exacerbated inflammation and imbalance in pregnancy-related molecules. This approach highlights the importance of performing comparative genomic analysis between congenital and high-virulence strains to identify the molecular mechanisms involved in adaptation during congenital transmission [87]. 

### 3.1. Enrichment Analysis of Transcriptomic Profiles Using DAVID

We analyzed three RNA-seq studies published between 2010 and 2024 to investigate transcriptomic insights into congenital Chagas disease (CD). Relevant articles were identified using keywords such as “RNA-seq”, “CONGENITAL CHAGAS DISEASE”, “TRYPANOSOMA CRUZI”, “TRANSCRIPTOMICS”, and “PLACENTAL”. The search algorithm used to identify these studies is outlined in Figure S1. Studies unrelated to congenital CD or those lacking data on differentially expressed genes (DEGs) were excluded from the analysis.

**Figure 3:**
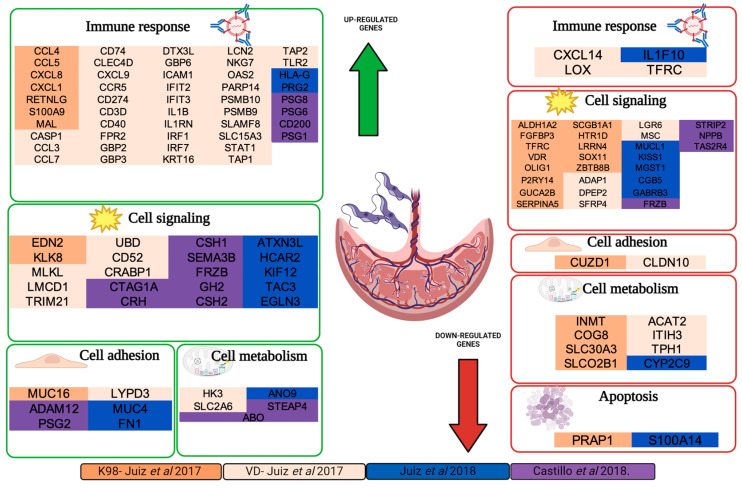
Functional categories of differentially expressed genes in the placental environment during *Trypanosoma cruzi* infection. This figure highlights the functional categories of differentially expressed genes (DEGs) involved in the interaction between *Trypanosoma cruzi* and the placental environment, which plays a critical role in the congenital transmission of Chagas disease. Up-regulated genes are shown in green boxes, while down-regulated genes are in red boxes, illustrating the biological processes activated or suppressed during infection. Background colors within the boxes correspond to the sources of the transcriptomic data, providing a clear link to the original studies and integrating findings across multiple investigations to offer a comprehensive overview of the key transcriptomic changes induced by *T. cruzi* [56,57,83].

The DEGs, along with their corresponding fold change values and cutoff points of >2 for up-regulated genes and <−2 for down-regulated genes, were extracted from the Supplementary Materials of the selected studies. These datasets were then processed using the DAVID tool for functional annotation and enrichment analysis. This approach enabled the identification of key biological pathways, cellular processes, and molecular functions associated with the congenital transmission of *T. cruzi*. The findings from these analyses provide valuable insights into the transcriptomic profiles of *T. cruzi* infection in both in vivo and ex vivo models of congenital transmission. Overall, the studies focused on identifying biological functions and metabolic pathways linked to the genes of interest (Figure 3).

In Juiz’s study, the up-regulation of genes involved in the immune response was evident, emphasizing the activation of innate immunity and the production of pro-inflammatory cytokines. Conversely, the K98 model displayed a predominance of secreted immune molecules and an intensified pro-inflammatory response characterized by the activation of pathways linked to host defense. Functional ontology analyses highlighted key processes, such as the immune response mediated by antimicrobial peptides, which are essential for the initial containment of pathogens.

Among these processes, interleukin-8 (IL-8) emerged as a pivotal pro-inflammatory cytokine that plays a critical role in neutrophil chemotaxis and activation. IL-8 also regulates essential functions, including neutrophil trafficking, cell adhesion, and placental development. In trophoblastic cells, IL-8 promotes migration and invasion while stimulating progesterone secretion. These cells rely on a finely tuned balance of cytokine signaling to maintain their functionality. However, dysregulation of these signals can result in pathological conditions such as pre-eclampsia [88]. These findings underscore the critical role of innate immune responses as the first line of defense against infections, with IL-8 playing a central role in regulating and coordinating these processes.

In VD, the over-regulation of immune processes is prominent, with inflammatory pathways playing a central role in its pathogenesis. Specifically, the cellular response to interferon type II (IFIT2) is crucial for macrophage activation and the promotion of microbicidal mechanisms. However, the overproduction of this mediator during the chronic phase can exacerbate inflammatory damage, contributing to the tissue deterioration associated with the disease [89,90]. Responses mediated by tumor necrosis factor (TNF-α) and interleukin-1 (IL-1) play a crucial role in balancing the containment of *T. cruzi* and tissue protection. While the activation of these cytokines is beneficial for controlling the infection, their dysregulation can lead to significant inflammatory damage. In this context, IL-1RN, an interleukin-1 receptor antagonist, regulates inflammation by preventing overexposure to inflammatory stimuli that could result in tissue damage [91,92,93]. This balance mediated by IL-1RN is essential for moderating the immune response, promoting both the control of the infection and the prevention of chronic inflammatory complications.

In line with this mechanism, an over-regulation of CASP1 was observed, a key protease in the activation of caspase-1, responsible for the maturation of interleukins IL-1β and IL-18, which are crucial for the immune response. As part of the inflammasome, CASP1 triggers the inflammatory response during infection, playing a critical role in defense against intracellular pathogens such as *T. cruzi*. In chronic Chagas myocardiopathy, T-cell-mediated inflammation and autoimmune mechanisms contribute to myocardial damage and dysfunction of the cardiac conduction system, highlighting the importance of the CASP1 pathway in the development of this disease [91,94].

Additionally, over-regulated processes such as intracellular calcium homeostasis and the regulation of GTPase activity were observed, both of which play fundamental roles in membrane trafficking and exocytosis. GTPases act as key regulators of various cellular processes, controlling vesicle transport from intracellular compartments. In collaboration with SNARE proteins, GTPases promote efficient membrane fusion during exocytosis. They also participate in calcium-dependent pathways, such as lysosomal exocytosis, to respond to damage in the plasma membrane [95]. This mechanism is linked to the invasion processes described by the parasite, in which it induces damage to the plasma membrane, triggering calcium-dependent lysosomal exocytosis [96,97,98]. These lysosomes fuse with nascent parasitophorous vacuoles, allowing the parasite to escape into the cytosol and transform into replicative amastigotes. This coordinated process emphasizes the crucial role of GTPases in maintaining cellular homeostasis and responding to the dynamic demands of the cellular environment. The parasite enhances its invasion and survival within host cells by modulating these processes.

On the other hand, another key component in the immune response was identified: signaling mediated by TLRs, which play an essential role in innate immunity by detecting various organisms, including bacteria, fungi, parasites, and viruses [99,100]. In humans, 10 functional TLRs are expressed not only in immune cells, such as macrophages, dendritic cells, and B lymphocytes, but also in non-immune cells, including fibroblasts and epithelial cells [101]. These receptors are crucial for the initial detection of *T. cruzi*, the recruitment of phagocytes, and the activation of inflammatory cytokines [102,103]. However, their dysregulated activation can contribute to the development of inflammatory and autoimmune pathologies, a phenomenon commonly observed in other parasitic and inflammatory diseases, such as leishmaniasis and tuberculosis [104,105,106]. This immune imbalance reflects shared mechanisms of tissue damage and underscores the immune modulation strategies employed by *T. cruzi* to evade the host’s response, leading to progressive tissue damage associated with chronic inflammation [107,108].

The findings reported by Juiz (2018) and Castillo (2018) highlight the presence of immunomodulatory processes and mechanisms regulating the placental microenvironment in the context of congenital CD. Among these, the overexpression of the HLA-G gene stands out. This gene encodes a non-classical molecule of the major histocompatibility complex (MHC) and plays a key role in maternal–fetal immune tolerance. In the literature, elevated levels of HLA-G in mothers infected with *Plasmodium* have been associated with increased susceptibility to infection. This phenomenon suggests that modulation of HLA-G could influence the progression of *T. cruzi* infection and the maternal immune system’s ability to control the disease and protect the fetus [56,83,109].

In Castillo’s study (2018), the over-regulation of genes from the PSG (pregnancy-specific glycoproteins) family, such as PSG1, PSG6, and PSG8, is reported. These genes play a key role in modulating the immune system during pregnancy. These molecules affect dendritic cells, promoting a balanced immune response that, on one hand, protects against infections, and on the other, supports the tolerance necessary for a successful pregnancy [110,111]. This dynamic suggests that *T. cruzi* could promote a tolerogenic environment that facilitates its congenital transmission, exploiting these maternal immune mechanisms to evade the host’s immune response and ensure its survival.

In the reviewed studies, the down-regulation of processes associated with cellular signaling is observed, suggesting *T. cruzi*’s intervention in various biological pathways. Among the proposed strategies, the following stands out.

### 3.2. Immune Evasion and Modulation of the Maternal–Fetal Immune Response by T. cruzi

The down-regulation of genes related to the immune response, such as SERPINA5, reflects *T. cruzi*’s ability to interfere with key processes of the immune system. SerpinA5, which is involved in various physiological roles, such as host defense, particularly against HIV, still requires further research to clarify its mechanisms of action in these contexts [112,113]. On the other hand, the vitamin D receptor (VDR), known for its significant impact on the immune system, plays a crucial role in modulating immune responses in various physiological and pathological processes [114]. The combination of the down-regulation of SERPINA5 and the disruption of vitamin D signaling suggests that *T. cruzi* employs complex strategies to modulate the activation of key immune mechanisms, promoting a tolerant environment that could facilitate its survival and transmission while evading the host’s immune response.

### 3.3. Disruption of Cellular Repair and Tissue Homeostasis

The down-regulation of genes such as OLIG1, which is essential for the differentiation and maturation of oligodendrocyte precursor cells, suggests that *T. cruzi* may interfere with cellular repair and tissue homeostasis mechanisms [115,116]. Additionally, SOX11, a key transcription factor in the regulation of nervous system development and cellular differentiation, particularly in myelination processes and injury response, indicates a disruption in crucial developmental and cellular repair processes [117]. Furthermore, FGFBP3, a protein essential for regulating fibroblast growth factors (FGFs), modulates key processes such as cell proliferation, differentiation, and migration. FGFBPs, like BP1, are critical for the activation of FGFs, regulating processes such as development, angiogenesis, and tissue repair [118]. The down-regulation of these genes, involved in cellular differentiation, tissue growth, and repair, could interfere with cellular regeneration mechanisms, impairing the host’s ability to repair damaged tissues.

### 3.4. Alteration of Hormonal and Metabolic Signaling

The down-regulation of the KISS1 gene, which encodes kisspeptin, a group of peptide fragments, has been implicated in regulating various key physiological processes such as placental angiogenesis and trophoblast invasiveness, modulating the activity of matrix metalloproteinases and promoting placental remodeling. Disruption of this pathway could contribute to pregnancy instability, as a decrease in kisspeptin expression has been observed in women with recurrent miscarriages. Furthermore, studies suggest that measuring serum kisspeptin levels could serve as a useful biomarker for predicting pregnancy viability [84,119,120]. In addition, the LGR6 gene plays a crucial role in regulating cellular differentiation and proliferation in stem-cell-dependent tissues such as bones and skin, highlighting its importance in tissue regeneration. Alterations in LGR6 expression can create an imbalanced physiological environment, promoting parasite adaptation while interfering with tissue homeostasis [121,122]. Both KISS1 and LGR6 play pivotal roles in regulating essential biological processes, such as placental angiogenesis, trophoblast invasiveness, tissue regeneration, and stem cell differentiation. The down-regulation of these genes suggests that *T. cruzi* may exploit these pathways to manipulate the host’s physiological environment, thereby promoting its survival and replication. By disrupting key processes such as hormonal signaling, cellular proliferation, and tissue homeostasis, the parasite may create a favorable environment that enhances immune evasion and supports congenital transmission. These findings indicate that *T. cruzi* employs multiple strategies to manipulate the host’s immune response and critical cellular processes, exploiting vulnerabilities to ensure its survival and transmission [123].

A decrease in metabolic, biosynthetic, and macromolecular transport processes has been observed. These alterations in infected and immune cells could arise from the dysregulation of various metabolic pathways. One example is COG8, which is essential for vesicular trafficking in the Golgi apparatus. This protein participates in the formation of SNARE complexes, which are crucial for retrograde transport between the Golgi and other cellular compartments, ensuring the proper localization of glycosylation enzymes in the Golgi. Furthermore, COG8 is involved in biogenesis and vesicular trafficking, highlighting its importance in maintaining cellular functionality [124,125]. Similarly, the transporters SLC30A3 and SLCO2B1 are involved in the transport of zinc from the cytosol to the extracellular space or subcellular organelles, as well as in the transport of steroid and thyroid hormones, which are essential for pregnancy and fetal development, respectively. The placenta regulates the transport of nutrients, hormones, and drugs through ABC and SLC transporters in the syncytiotrophoblast. According to the literature, placental inflammation can alter the expression of these transporters, suggesting direct interference in nutrient uptake and intracellular transport functionality, thus affecting their activity and fetal exposure. This phenomenon has been reported in cases of premature births. Moreover, alterations in CYP2C9, which plays a key role in the metabolism of xenobiotics and endogenous molecules, could reflect an impact on the host’s adaptive metabolic capacity [126]. These conditions not only compromise the normal metabolic balance of cells but also affect the regulation of essential hormones and molecules during pregnancy, potentially having implications for fetal development and pregnancy-related complications [127,128].

Additionally, the down-regulation of apoptosis-related genes was observed, such as PRAP1 (Proline-rich acidic protein 1), which plays a crucial role in protecting cells from damage induced by oxidative stress and radiation. Its main function appears to be activation in response to DNA damage, intervening in the p53 signaling pathway to protect cells and improve their viability [129]. On the other hand, S100A14, which belongs to the S100 protein family—small calcium-binding proteins—acts as both an intracellular and extra-cellular signaling molecule. These proteins modulate a wide range of intracellular functions by altering their subcellular localization and interacting with specific target proteins involved in cell growth, differentiation, motility, apoptosis, and cell cycle regulation [130]. This information suggests direct interference with programmed cell death mechanisms. This phenomenon may represent a strategy employed by the parasite to prevent the elimination of infected cells, thereby ensuring its survival in the maternal–fetal environment. Inhibition of apoptosis would create a cellular environment favorable for parasite replication while impeding the host’s ability to remove damaged or infected cells. Collectively, these processes could help explain clinical observations such as fetal growth retardation in mothers infected with *T. cruzi* and the emergence of gestational disorders like pre-eclampsia [131].

In total, these factors contribute to the disruption of the delicate molecular balance in the chorionic villi, leading to destabilization and loss of structure, thereby creating an environment conducive to the parasite’s invasion. These changes, combined with the down-regulation of apoptotic processes, not only facilitate congenital transmission but also enable the persistence of the parasite within the tissue.

## 4. MicroRNAs and Their Role in Post-Transcriptional Regulation During Parasitic Infections

To understand the underlying molecular mechanisms in the pathophysiology of the disease and the complex interactions between *T. cruzi* and its host, it is crucial to consider the parasite’s ability to modulate host gene expression through non-coding RNAs, such as microRNAs (miRNAs). These miRNAs play a fundamental role in the post-transcriptional regulation of mRNA and protein expression, influencing the molecular interaction networks that govern the host’s response to infection [132]. Additionally, miRNAs are being investigated as non-invasive biomarkers due to their easy detection in biological samples, offering significant advantages in terms of accessibility and diagnostic accuracy [133,134].

In humans, chromosome 19 contains the majority of miRNAs, many of which are specifically expressed in the placenta, underscoring their importance in tissue-specific expression profiles [135]. These miRNAs are essential in critical cellular processes during infection, including development, cell proliferation and differentiation, apoptosis, metabolism, and modulation of the immune response [136,137].

A study by Medina et al. (2020) [138] examined the effects of *T. cruzi* and Toxoplasma gondii on the miRNA profile in human placental explants, revealing significant overexpression of three miRNAs: miR-21, miR-146a/b, and miR-210. miR-21, which plays a role in cell proliferation, differentiation, migration, and apoptosis regulation, showed increased expression, potentially linked to physiological changes during pregnancy. The down-regulation of miR-21 in the placenta has been associated with suboptimal fetal growth and may have important implications for fetal development [139,140].

Regarding miR-210, differential expression was observed between normotensive placentas and those with severe pre-eclampsia, suggesting its role as a mediator of mitochondrial dysfunction in pre-eclampsia and a potential marker of hypoxia [141]. In the context of *T. cruzi* infection, miR-210 was overexpressed in infected explants, likely due to the activation of NF-kB pathways, which regulate the transcription of this miRNA [138].

On the other hand, miR-146a, which is induced by NF-kB, plays a crucial role in regulating the placental inflammatory response by preventing dysregulated inflammation. Down-regulation of this miRNA promotes the invasion of *T. cruzi* and *T. gondii*, resulting in an increased parasitic load [142,143]. Moreover, a notable overexpression of miR-512-3p was observed, which inhibits the caspase 8 inhibitor (c-FLIP), thereby enhancing caspase 8 activity. This up-regulation of caspase 8 activity could act as a protective mechanism against infection by promoting the removal of infected or damaged cells, thereby minimizing potential adverse effects on pregnancy [70,138].

In a further analysis, Medina et al. (2022) [144] examined the placental response to *T. cruzi* infection, revealing alterations in miRNA profiles that closely resembled those observed during trophoblast differentiation. The study focused on two miRNAs, miR-512-3p and miR-515-5p, which have opposing effects. While the up-regulation of miR-512-3p promotes cellular turnover, the up-regulation of miR-515-5p inhibits differentiation by directly repressing the transcription factor hGCM-1. miR-515-5p has been proposed as a potential biomarker for pre-eclampsia when overexpressed [145]. It was found that the positive regulation of miR-512-3p and the negative regulation of miR-515-5p promote cell turnover and differentiation, influencing the balance of local defense mechanisms in the placenta against the parasite.

In contrast, inhibition of miR-512-3p and increased levels of miR-515-5p resulted in an increase in parasite DNA load. This suggests that by interfering with trophoblast differentiation through these two miRNAs, one of the initial innate immunity mechanisms in the placental layer is compromised [144]. It is essential to note that the interactions between these molecules are not yet fully understood, and they may work synergistically or regulate other established processes. Therefore, it is crucial to conduct targeted studies to identify the specific microRNAs involved in *T. cruzi* infection and clarify their regulatory roles in this context. These investigations will offer a deeper understanding of the placental response mechanisms to the infection.

## 5. Future Directions

It is essential to conduct studies that deepen our understanding of the pathogenesis and host response during congenital *T. cruzi* infection, focusing on transcriptomic, proteomic, and metabolomic aspects. Transcriptomic studies are particularly valuable as they provide insights into the changes in gene expression during congenital transmission. These studies offer a detailed understanding of the regulatory mechanisms and molecular pathways involved in the host’s response, which can guide subsequent proteomic and metabolomic investigations [146,147].

Integrating protein and metabolite profiles will help clarify how the products of these transcriptomic changes regulate immune responses, and the biological processes involved in transmission. Taken together, these multidisciplinary approaches will not only provide a more comprehensive view of congenital CD pathogenesis but also reveal new opportunities to identify therapeutic targets and biomarkers and develop targeted intervention strategies [81].

However, designing these studies requires careful consideration of the complexity and multifactorial nature of this transmission mechanism. Various factors must be thoroughly evaluated, as they may significantly impact the results and interpretation of the findings. One crucial factor is the selection of the *T. cruzi* genotype. With multiple strains exhibiting variations in virulence, pathogenicity, and tissue tropism, interactions with host cells can differ, affecting immune signaling and response [57,148]. Therefore, selecting the appropriate genotype that mirrors the key characteristics of congenital infection is essential for obtaining relevant and applicable results.

Furthermore, choosing the correct animal or cell model is critical, as different models can replicate specific aspects of congenital infection, providing valuable insights into the mechanisms at play [149,150]. Each model has its strengths and limitations, and the choice should align with the study’s objectives. Primates are particularly valuable due to their close resemblance to human placental structures, making them ideal for studying the pathogenesis of congenital transmission [24,151]. However, ethical concerns and high maintenance costs often limit their use. Domestic dogs, while having a distinct type of placentation, are more accessible and offer opportunities to investigate transmission dynamics and immune responses [152]. These species also serve as potential reservoirs, especially in endemic regions, emphasizing their relevance in research.

Rodent models, particularly mice and guinea pigs, are commonly used to study congenital transmission of *T. cruzi*. In mice, infection during pregnancy results in fetal malformations and growth retardation, with the parasite detectable in fetal tissues [153,154,155]. However, mice are not ideal for studying trophoblast invasion and vascular remodeling, which are critical in fetal growth restriction and pre-eclampsia in pregnant women [156]. Guinea pigs, with a longer gestation period and more human-like fetal development, offer a more suitable model. Infected guinea pigs transmit *T. cruzi* to all offspring, leading to tissue damage and growth retardation, with PCR confirming the presence of the parasite in both the placenta and fetuses [157,158].

The structural and physiological differences in placentas among larger mammals provide valuable opportunities for comparative research, supporting studies of human infection and epidemiological investigations in regions where these species serve as reservoirs or are in close contact with humans [24]. Incorporating advanced molecular techniques like transcriptomics and proteomics enhances understanding of *T. cruzi* interactions with the host during gestation, revealing critical processes such as immune evasion and placental remodeling that facilitate transmission. The genetic variability of *T. cruzi* strains, including Discrete Typing Units (DTUs) like TcI, TcII, and TcV, influences infectivity, virulence, and tissue tropism—key factors in congenital transmission. Including these diverse DTUs in experimental studies allows researchers to explore strain-specific interactions with the host and placenta, providing deeper insights into the epidemiological patterns of congenital Chagas disease and the factors driving its transmission.

Another key consideration is the selection of exposure times and parasitemia levels. Variations in these factors can affect infection dynamics and the molecular and cellular responses [159]. Therefore, establishing experimental protocols that accurately reflect the conditions of congenital transmission is essential. Finally, the choice of omics analysis is also critical. This review emphasizes the value of dual RNA-seq analysis, which enables the evaluation of gene expression modulation in both the host and the pathogen, providing a comprehensive picture of signaling and virulence factors involved in congenital transmission [160,161]. This approach also allows for the examination of differences in invasion and signaling mechanisms, which may differ in various tissues, and helps identify key factors contributing to the development of congenital CD.

Once these key elements are identified, validation through proteomic tools can further solidify the findings, providing a foundation for selecting therapeutic targets and developing strategies to interrupt transmission.

The development of single-cell analysis has gained significant relevance due to its ability to reveal hidden cellular heterogeneity, which is overlooked by traditional approaches that analyze combined cell populations. These variations play a crucial role in the diagnosis and treatment of diseases, enabling the identification of specific responses to therapies [162,163]. Single-cell technologies, by allowing highly sensitive investigations, are fundamental for personalized medicine and more precise diagnoses. In the case of congenital Chagas disease, this approach facilitates the study of the interaction between trophoblasts and *T. cruzi*, uncovering key mechanisms in disease progression, such as immune evasion and placental modulation, thus opening new therapeutic avenues.

Moreover, there is a need to further explore additional regulatory elements, such as non-coding RNAs, which play essential roles in post-transcriptional regulation and contribute to fundamental biological pathways during gestation and infection. MicroRNAs may play a pivotal role in the pathophysiology of congenital Chagas disease transmission. Identifying and characterizing the specific miRNAs involved could provide valuable insights into the molecular mechanisms and signaling pathways at play. These microRNAs could also serve as potential biomarkers for early diagnosis, prognosis, and severity assessment of congenital infection.

## Data Availability

Not applicable.

## Supplementary Materials

The following supporting information can be downloaded at: https://www.mdpi.com/article/10.3390/pathogens14020106/s1. Figure S1. Review for identifying and categorizing research articles on congenital Chagas disease with a focus on RNA-seq and transcriptomic data. This algorithm outlines a review approach used to identify and categorize research articles related to congenital Chagas disease, specifically focusing on RNA-seq and transcriptomic data. The process includes retrieving studies from databases like PubMed, excluding non-congenital research, and analyzing articles that provide data on differentially expressed genes (DEGs), including available Fold Change values.
