# Supplementary material for: Understanding Host–Pathogen Interactions in Congenital Chagas Disease Through Transcriptomic Approaches
Source: Pathogens. 2025 Jan 22;14(2):106. doi: 10.3390/pathogens14020106 (PMC11858232; doi:10.3390/pathogens14020106)
Supplement: Supplementary file 1 [file pathogens-14-00106-s001.zip › pathogens-3403357-supplementary.pdf]

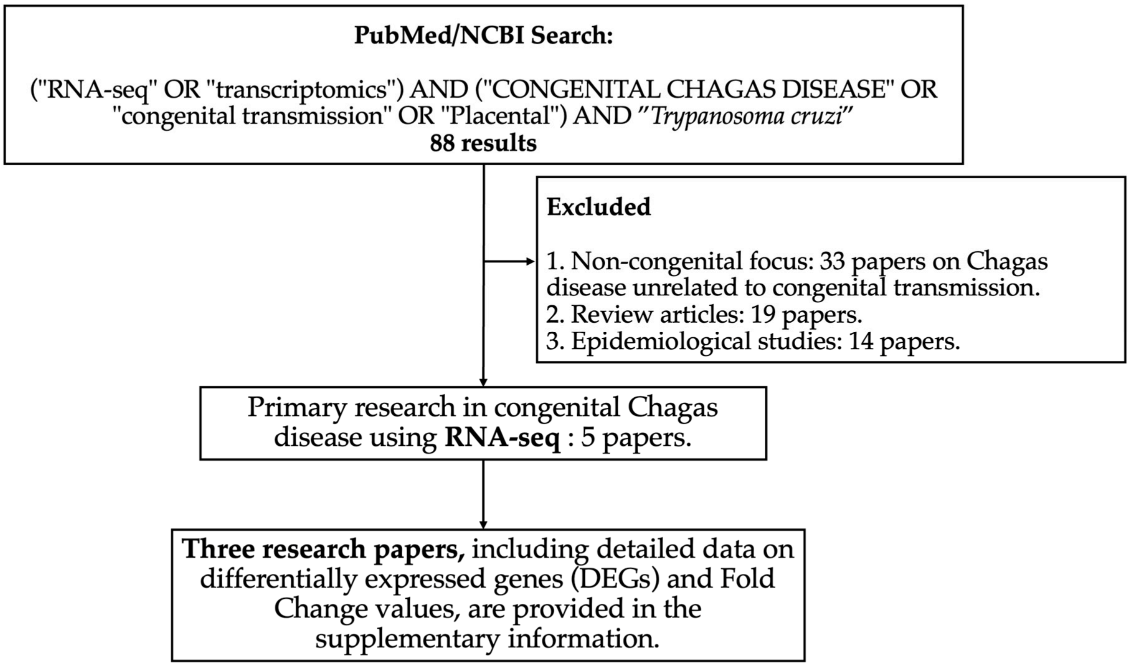

Figure S1. Review for identifying and categorizing research articles on congenital Chagas disease with a focus on RNA-seq and transcriptomic data. This algorithm outlines a review approach used to identify and categorize research articles related to congenital Chagas disease, specifically focusing on RNA-seq and transcriptomic data. The process includes retrieving studies from databases like PubMed, excluding non-congenital research, and analyzing articles that provide data on differentially expressed genes (DEGs), including available Fold Change values.
